# Effect of Energy Density on the Mechanical Properties of 1.2709 Maraging Steel Produced by Laser Powder Bed Fusion

**DOI:** 10.3390/ma17143432

**Published:** 2024-07-11

**Authors:** István Hatos, Hajnalka Hargitai, Gusztáv Fekete, Imre Fekete

**Affiliations:** Department of Material Science and Technology, Audi Hungária Faculty of Vehicle Engineering, Széchenyi István University, H-9026 Győr, Hungary; hatos@sze.hu (I.H.); hargitai@sze.hu (H.H.); fekete.imre@sze.hu (I.F.)

**Keywords:** additive manufacturing, impact energy, energy density, porosity, 1.2709 steel

## Abstract

The unusual combination of the fundamentally contradictory properties of high tensile strength and high fracture toughness found in maraging steel makes it well suited for safety-critical applications that require high strength-to-weight materials. In certain instances, additive manufacturing (AM) has produced materials that may be desirable for safety-critical applications where impact toughness is a key property, such as structural parts for the aerospace industry or armor plates for military applications. Understanding the influence of process parameters and defect structure on the properties of maraging steel parts produced via laser powder bed fusion (LPBF) is a fundamental step towards the broader use of AM technologies for more demanding applications. In this research, the impact energy of V-notched specimens made of 1.2709 maraging steel produced by LPBF was determined via Charpy impact testing. Specimens were produced using different processing parameter sets. By combining the process parameters with the porosity values of the parts, we demonstrate that an almost full prediction of the impact properties can be achieved, paving the way for significantly reducing the expenses of destructive testing.

## 1. Introduction

Layer-manufacturing methods first appeared in the late 1980s. Currently, this technology is used in several industries, where various processes use “layer manufacturing”. The scientific and practical literature identify this technology with different denominations, such as 3D printing, additive manufacturing (AM), direct digital manufacturing, and rapid prototyping [1,2]. Laser powder bed fusion (LPBF) processing is the most common AM technology used to produce metal parts. Selective laser melting (SLM) and direct metal laser sintering (DMLS) are the main two LPBF processes. Both use a focused laser beam to melt metallic powder particles together with respect to the layer geometry. Due to the ability of the LPBF process to produce advanced 3D parts with high strength and toughness, it has found applications in various sectors, such as the military [3], die casting [4], the energy industry [5], and dental applications [6,7], as well as for producing general purpose mechanical parts [8,9].

The variation of individual parameters affects the final quality of the workpiece, the cost of production, and the duration of the 3D printing. In general, the initially set production parameters are kept constant during laser melting. Improper printing parameters can lead to unwanted porosity, print instability, and poor mechanical properties. The main parameters include the layer thickness, scanning speed, laser power, hatch spacing, scanning direction, and strategy [10]. The process parameters can be simplified with a theoretical index known as the volumetric energy density (VED):

(1) $$E=\frac{P}{vht}$$

This index is used to summarize the energy level per unit volume (J/mm^3^) considered during layer melting. *P* (W) denotes the laser power, *v* (mm/s) denotes the laser scanning speed, *h* (mm) denotes the hatch spacing, and *t* (mm) denotes the layer thickness [11].

Although LPBF intends to produce nearly fully dense parts, the incorrect selection of process parameters often results in the formation of pores. Process-induced porosity remains one of the major concerns in metal additive manufacturing [12]. In the literature, three methods are used to determine the porosity of AM parts: direct measurement of density using Archimedes’ method, image analysis on a micrograph, and computed tomography (CT) [13]. Wits et al. [14]. focused on comparing porosity-testing methods, where the experimental results showed that the results of Archimedes’ method are comparable to the CT results, although CT measures smaller values of porosity (percentage for volume) than microscopic methods.

Three types of pores can be formed during LPBF manufacturing: gas, keyhole, and lack of fusion (LoF).

Gas pores are the most common pore type in AM. These pores are the smallest and most spherical. 

Keyhole pores are caused by over-melting due to high energy-density values. In practice, these pores appear when high beam power is used, leading to excessive penetration, which leaves a pore near the bottom of the melt pool after solidification [15]. 

LoF pores are caused by a lack of input energy, which means that the absorbed energy is insufficient to fully melt the powder, resulting in large and irregular porosities. 

**Figure 1:**
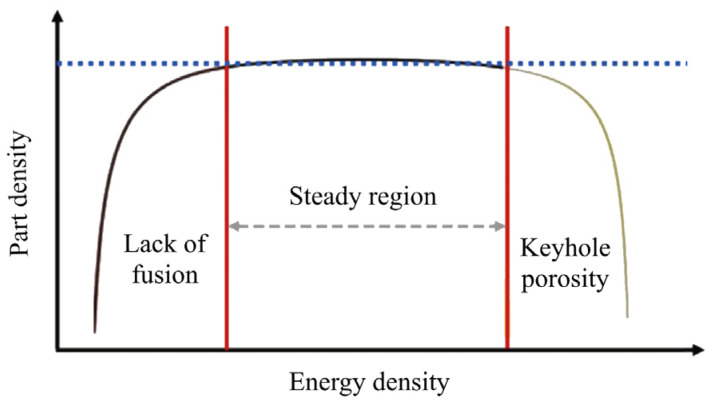
Relationship between part density and energy density [16].

By combining various process parameters, it is possible to identify a steady region (the region between high and low energy density) where optimal processing conditions can be utilized to achieve good mechanical properties (Figure 1) [16].

It must be noted that the VED has an optimal range of 70–185 J/mm^3^ [17], where highly dense 1.2709 parts (relative density in the range of 99–99.99%) can be achieved. Mugwagwa et al. [18] analyzed the influence of laser power, scanning speed, and layer thickness on the density of 1.2709 parts in order to establish optimal parameter combinations. It was found that high porosity occurs due to both overheating and insufficient heating. The best relative densities were found in samples manufactured at 600 mm/s and 180 W. A similar optimum value (laser power = 165 W and scan speed = 784 mm/s) was determined by Maodzeka et al. [19]. In LPBF processing, the metal powder is spread in a layer with a certain layer thickness, typically in the range of 20–100 µm [20]. During another experiment [21], the process parameters for production with a layer thickness of 100 µm were investigated. The best result of 99.982% relative density was obtained using 340 W and 500 mm/s printing parameters.

Maraging steel is well-suited for applications where high strength and fracture or impact toughness are key properties, such as in armor plates, tools, and landing gears [22]. Several studies have examined the influence of processing parameters on the tensile properties of AM 1.2709 parts [22,23]. In contrast to the tensile properties, according to the authors’ knowledge, the impact behavior of 1.2709 components produced by AM has rarely been analyzed. Knowledge of the ssimpact behavior is fundamental to several applications where rapid loading conditions are present. Impact energy is a measure of the amount of energy absorbed; it is usually determined through the Charpy impact test [24]. Jarfors et al. [25] studied the impact properties of LBPF-manufactured 1.2709 maraging steel. Test samples were produced in vertical and horizontal building directions. They used a zig-zag pattern rotated by 67° on every layer. There were no significant differences between the measured values in different building directions. The effect that porosity plays on the tensile and toughness properties of Ti-6Al-4V parts was investigated as well [26]. The authors reported that increasing the porosity decreased the impact toughness and noted that regions subjected to impact loading should be built with highly dense parameters. 

This paper aims to expand these studies by testing a series of 24 process parameter sets to determine the relationship between process parameters and impact energy. This study also aims to determine the parameters needed to achieve high strength and toughness.

## 2. Materials and Methods

Steel 1.2709 is a type of martensitic tool steel. Other international designations refer to it as 18Ni (300 grade) maraging steel, or X3NiCoMoTi 18-9-5. The powder used for this experiment was MS1 from EOS GmbH. The chemical composition of the 1.2709 powder was 17–19% Ni, 8.5–9.5% Co, 4.5–5.2% Mo, 0.6–0.8% Ti, 0.05–0.15% Al, <0.5% Cr, <0.1% Mn, <0.1% Si, <0.03% C, and balanced Fe (all in wt.%). The powder used for the specimens was reclaimed from prior jobs with the use of an 80 μm sieve. An EOSINT M270 machine (200 W, Yb-fibre laser, 1070 nm, and 100 μm focus diameter, EOS GmbH, Krailling, Germany) was used with nitrogen gas atmosphere.

**Figure 2:**
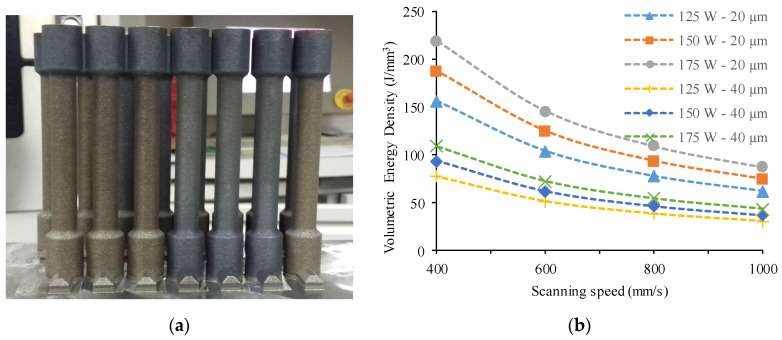
(**a**) Tensile test bars; (**b**) volumetric energy density variations against scanning speed.

Charpy impact test specimens and cylindrical Ø8 mm tensile test bars (Figure 2a) were built in the vertical direction using laser power levels of 125, 150, and 175 W; scanning speeds of 400, 600, 800, and 1000 mm/s; and layer thicknesses of 20 and 40 μm. Figure 2b shows the calculated energy density against the scanning speed. A stripe hatching strategy with 67° scanning rotation between subsequent layers was maintained during the process. The contouring parameters were deactivated so that only the hatching parameters were considered. All of the samples were made at a 40 °C building platform temperature, and the hatch distance was 0.1 mm.

**Figure 3:**
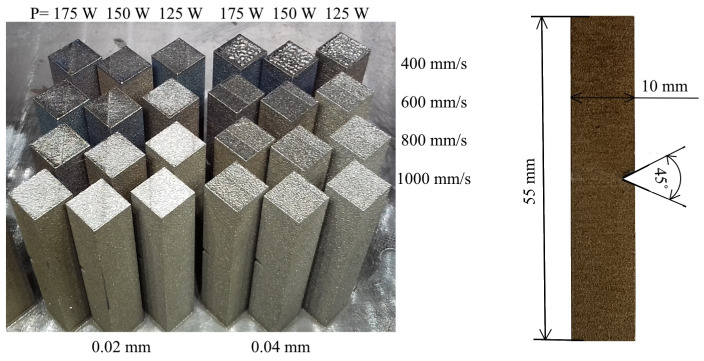
Charpy impact samples just after the LPBF process.

The Charpy samples had square cross-sections of 10 mm × 10 mm and lengths of 55 mm (Figure 3); they also contained 2 mm deep notch cut out with a 0.25 mm root radius at the center. Charpy impact tests were performed on V-notched specimens according to ASTM E23 standards [27] at room temperature. The nominal energy of the striker impact testing machine was 300 J.

Tensile test measurements were carried out on an INSTRON 5582 testing machine (INSTRON, Kawasaki, Japan) at room temperature using a non-contact video extensometer to measure strain. Tensile tests were conducted until break using displacement control at a rate of 10 mm/min. In our study, three specimens for each parameter set were tested, and from the load-extension curve, the yield strength, ultimate tensile strength, and percentage elongation after fracture (A_40_) were determined. 

The impact test samples were used for mechanical testing and microstructural characterization. The samples were prepared following standard metallographic practices described in one of our previous works [28]. The porosity of each sample was determined via an analysis of the metallographic longitudinal cross-sections parallel to the building direction. Prior to the microstructural examination, the samples were first ground with SiC grinding paper, then polished. A Zeiss Axio Imager M1 optical microscope with a motorized platform was used to image the prepared specimens. The determination of the (2D) porosity of the constant-sized regions was carried out via the following process. A grayscale image of the mosaic was created in order to make the material–void segmentation easier. A histogram of this image was used to determine a threshold value for the segmentation. This threshold value was chosen to separate the dark and light pixels (void and material, respectively). After segmentation, the area of voids and the area of material were calculated. The 2D porosity was calculated as the ratio of void area to total measurement area.

The specimens were investigated by Computed Tomography (YXLON Modular Y.CT system equipped with a 225 kV micro-focus X-ray tube and a Y.XRD1260 flat panel detector) to assess the internal structures. Tube voltage was 190 kV, and the tube current was 0.12 mA. The flat panel detector was set to operate in a 2 × 2 binning mode with an integration time of 1000 ms; 1440 projections were taken with no filters used. The small size of the specimens allowed good magnification, yielding a voxel size of 8.23 μm. The resulting 1024^3^ voxel data was loaded into VGStudio MAX for analysis. After the registration of the voxel dataset into a coordinate system, automated volumetric measurements were run.

The fracture surfaces of the Charpy impact specimens were surface scanned with GOM ATOS digital optical system. In order to measure the fracture surface area of the specimens the surface mesh was evaluated with GOM Inspect and Materialise Magics 27.0 software.

## 3. Results and Discussion

**Figure 4:**
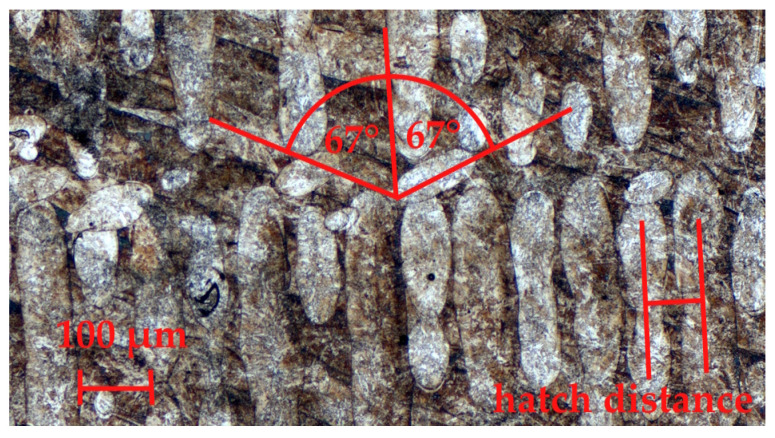
Optical micrographs of printed 1.2709 steel along build plane.

Maraging steel is well-suited for applications where high strength and fracture or impact toughness are key properties. For this reason, we investigated the effect that porosity plays on the tensile and toughness properties of 1.2709 parts. The impact energy, tensile properties, and relative density of each sample were evaluated. A micrograph of a printed parts along the build plane is shown in Figure 4. The build plane, which is perpendicular to the build direction, shows the pattern of hatching. The weld strips indicate the molten paths produced by the laser. The build plane micrograph reveals the presence of three hatch layers. The angle between layers was kept at 67°.

**Figure 5:**
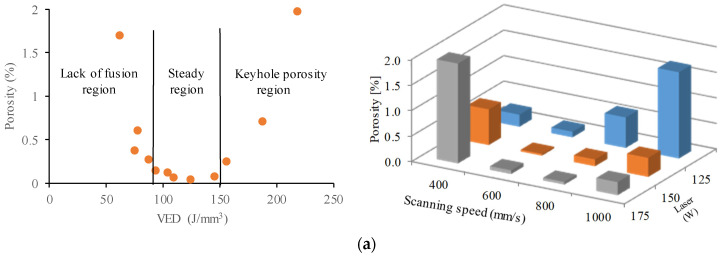

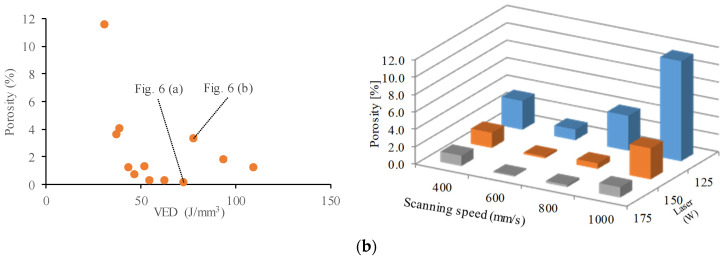
Relative porosity dependent on process parameters: (**a**) Layer thickness = 20 µm; (**b**) layer thickness = 40 µm.

The porosity distribution of the printed specimens with layer thicknesses of 20 µm are shown in Figure 5a. It can be noted that dense samples can be produced easily. Furthermore, this alloy followed the typical trend observed in most of the alloys processed by LPBF. The density increased with the increase of VED up to a specific value; however, further increase in VED resulted in lower density parts.

The VED values between 109.4 and 145.8 J/mm^3^ produced near fully dense samples. Overall, the results in Figure 5a suggest the VED range for stable conduction melting is from 90 to 150 J/mm^3^; the process parameter sets generated a porosity level below 0.25%.

**Figure 6:**
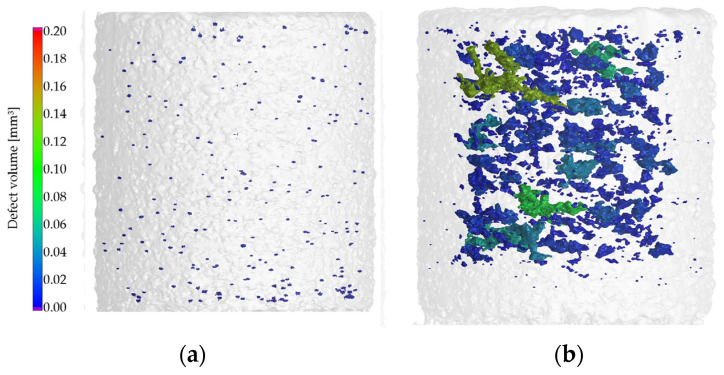
3D CT images with porosity analysis in the case of 40 µm layer thickness samples: (**a**) VED = 72.9 J/mm^3^; (**b**) VED = 78.1 J/mm^3^.

In the case of 40 µm layer thickness (Figure 5b), low porosity values could also be achieved. A greater layer thickness required more laser energy input to achieve a complete melting of layers, resulting in a higher penetration depth. Generally, VED is the main measure to determine the amount of energy imparted to the powder bed and the part. Samples with the same VED did not always exhibit the same amount of porosity. For example, in the case of the specimen 18, a VED of 72.9 J/mm^3^ (P = 175 W, v = 600 mm/s) was used that yielded a low amount of porosity of 0.11%. Furthermore, a small number of round voids can be observed, indicating a stable melt pool and low porosity (Figure 6a). These two indications point to a conduction mode melt behavior. On the other hand, specimen 13 was processed with almost the same VED of 78.1 J/mm^3^ (P = 125 W, v = 400 mm/s) but at a lower speed and laser power, resulting in a comparatively high 3.3% porosity. The 3D CT images of the porosity analysis of this specimen reveal that the voids are very large and numerous (Figure 6b). In the case of this specimen, the melt pool behavior is unstable. At low scanning speeds (400 mm/s), the outer surfaces of the specimens were dark regardless of the improper energy input. In case of over-heating, the temperature of the molten pool could reach the boiling point of the alloy, causing vaporization; pores in this sample are typical metal vapor pores. The above results demonstrate that VED is a simplified metric for LPBF; the individual printing parameters have great influence on the solidification. LPBF parameters of the same energy density could have yielded different melt-pool dimensions resulting in different-shaped pores within the parts. At higher layer thickness, the optimal processing window was narrower. 

**Figure 7:**
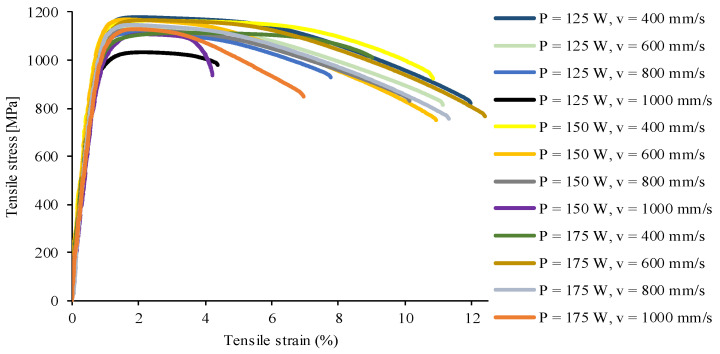
Tensile stress–strain curves for 1.2709 maraging steel.

The tensile mechanical responses of the 1.2709 steel printed at laser power levels of 125, 150, and 175 W; scanning speeds of 400, 600, 800, and 1000 mm/s; and layer thicknesses of 20 µm; shown in Figure 7.

**Figure 8:**
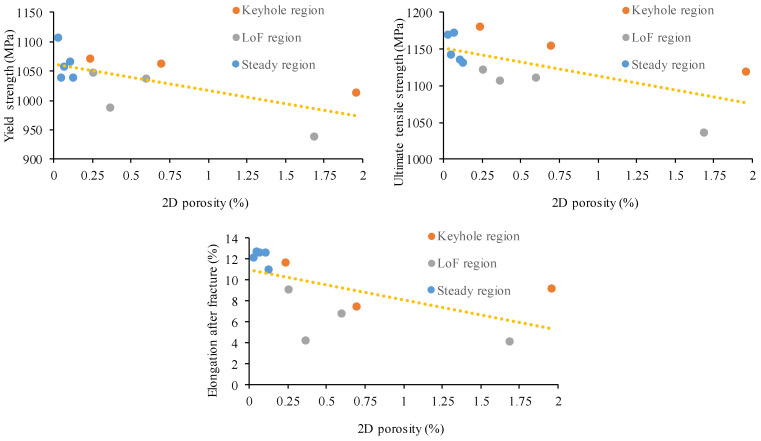
Summarized tensile properties of LBPF printed 1.2709 steel versus porosity fraction.

Three parallel tensile measurement results were used to calculate tensile properties. The obtained data are supplemented by the assessment of the influence of porosity on the tensile strength, yield point, and elongation of samples (Figure 8). Based on the results, it can be stated that the yield strength, tensile strength, and elongation of samples are effected by the porosity. Figure 7 reveals that the Young’s modulus of the material does not depend on the processing parameters.

This observation was confirmed in the scientific literature regarding LPBF alloys [13,26,29,30], which states that with increasing porosity, the tensile behavior ordinarily declines, which clearly indicates the connection between the defects in the structure and the mechanical properties of the metal. However, at low porosity levels, there may be no correlation between tensile properties and porosity. As the porosity nears zero, the effects of voids are diminished, and microstructural properties become the deciding factor during tensile loading.

**Figure 9:**
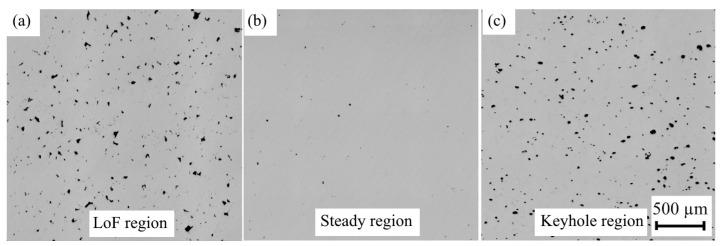
Porosity at different energy densities: (**a**) 87.5 J/mm^3^; (**b**) 109.4 J/mm^3^; (**c**) 218.8 J/mm^3^.

The upper and lower range of VEDs resulted in elevated porosity. The former condition leads to keyhole-induced pores while the latter leads to LoF voids. Furthermore, processing parameters resulting in LoF- and keyhole-type defects seem to display marked differences in yield and ultimate tensile strengths as well as elongation at break values. LoF-type defects tend to lower these properties compared to the keyhole-type defects. This is likely because the shape of LoF-type defects is sharp and their size can be large, which weakens the material more than the round and small keyhole-type defects. The keyhole pores are nearly spherical; in contrast, LoF voids can be any size and irregularly shaped (Figure 9). Based on the results, the shape of the porosity influences the tensile properties. At similar porosity levels, the presence of LoF pores leads to a measurable loss of tensile properties. The partially sharp-edged voids can act as stress concentrators, which can negatively influence the mechanical properties.

Regarding toughness, it is known that the presence of porosity and reductions in ductility can significantly reduce toughness [26]. The results of impact and porosity measurements are shown in Table 1. The maximum density and absorbed energy values achieved were 99.97% and 119 J for sample 5. When comparing this result with the highest-impact energy values of as-built LPBF 1.2709 specimens available in the literature [31], an improvement of more than 20% was achieved. The lowest recorded values of impact energy in this study were 10 J for 20 µm and 6 J for the parts with layer thicknesses of 40 µm.

**Figure 10:**
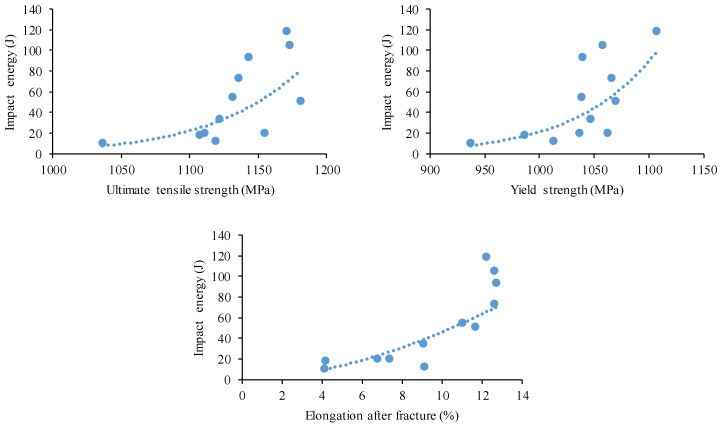
Variation in impact energy with YTS, UTS, and elongation.

Figure 10 shows the variation in impact energy as a function of yield strength, tensile strength, and elongation at break. In general, the impact toughness increased with increasing yield strength, tensile strength, and ductility. The larger scatter in the data predicted that no single parameter would exclusively predict any increase or decrease in toughness; this also depended on other parameters, such as the microstructure.

**Figure 11:**
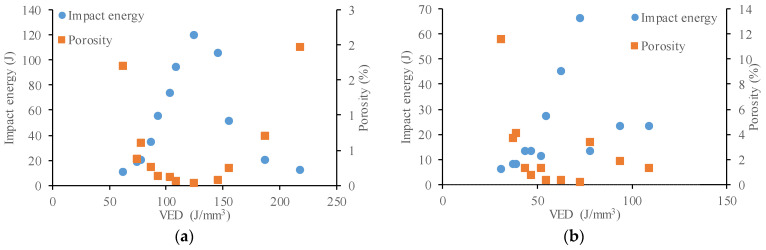
Effect of energy density on impact energy and porosity: (**a**) Layer thickness = 20 µm; (**b**) layer thickness = 40 µm.

The effect of the laser energy density on the impact energy was also investigated. An inverse relationship was found between the porosity and the impact energy of the specimens. This relationship can be observed in Figure 11, where high porosity specimens have low impact energies. In addition, nearly fully dense parts (low porosity) perform very well in impact testing. Based on these results, there is clear evidence that the porosity influences the toughness, and that porosity and toughness have a close relationship.

**Figure 12:**
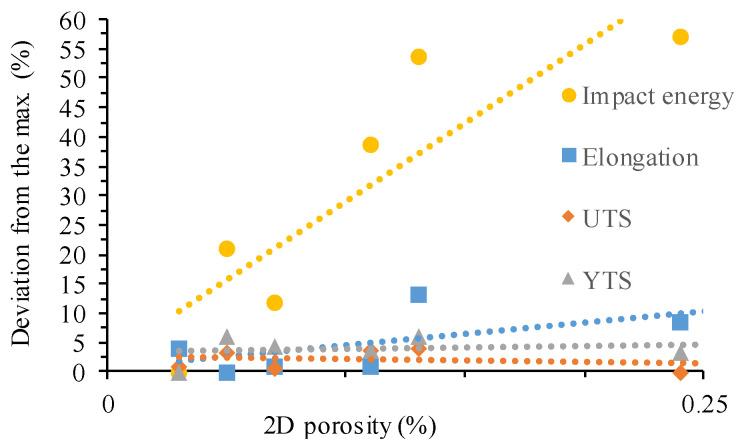
Effect of porosity on tensile and impact results.

Figure 12 shows the sensitivity of tensile properties and impact energy to porosity. At low porosity levels, the amount of porosity did not influence the yield or the ultimate strength results; thus, the effect of porosity is considered negligible. As can be seen from the results, the elongation at fracture was more sensitive to the porosity level as compared to the strength. This phenomenon can be explained by the failure mechanism of ductile materials, which begins with void nucleation followed by growth with increasing hydrostatic stress, local plastic straining, and then coalescence. The porosity at low concentrations does not lead to a measurable loss of stiffness, yield strength, or tensile strength, but does reduce the ductility, since microvoids exist before any stress is applied. The porosity has a high influence on the impact toughness. Herein, an increase in porosity drastically reduced the amount of absorbed energy. Impact testing could be used to optimize parameters because it is more sensitive to porosity changes and much faster compared to tensile testing.

**Figure 13:**
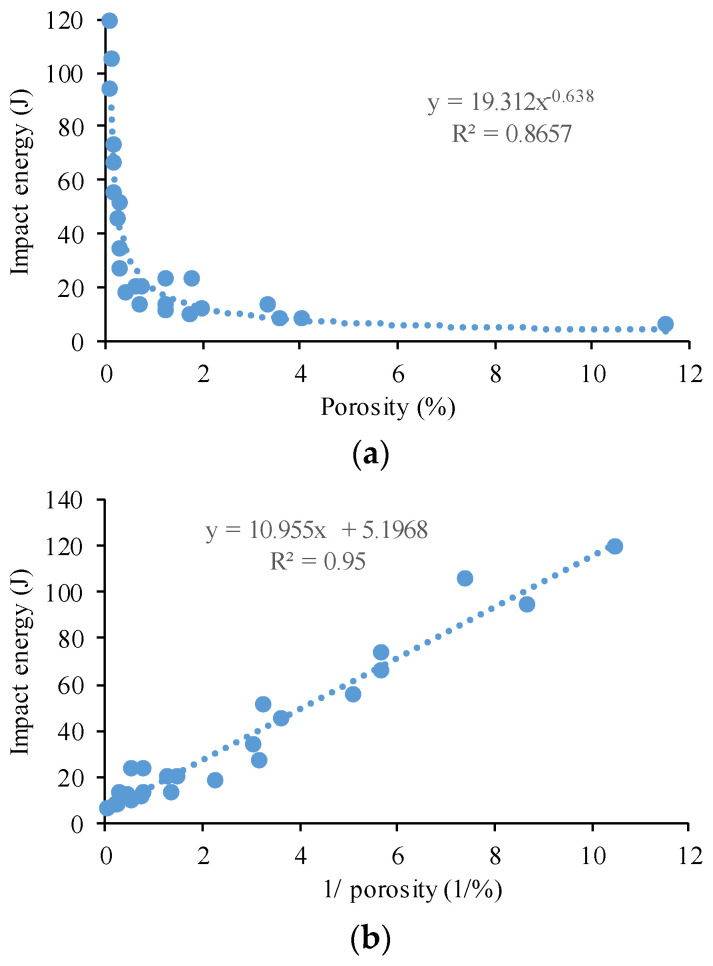
Absorbed energy as a function of: (**a**) porosity; (**b**) reciprocal of porosity.

The relationship between the porosity and the impact energy is shown in Figure 13. A strong linear relationship was found between the Charpy impact energy and the inverse of porosity. The goodness of fit (R^2^) of the linear correlation was 0.95. The more porosity exists in the material, the lower the absorbed energy.

**Figure 14:**
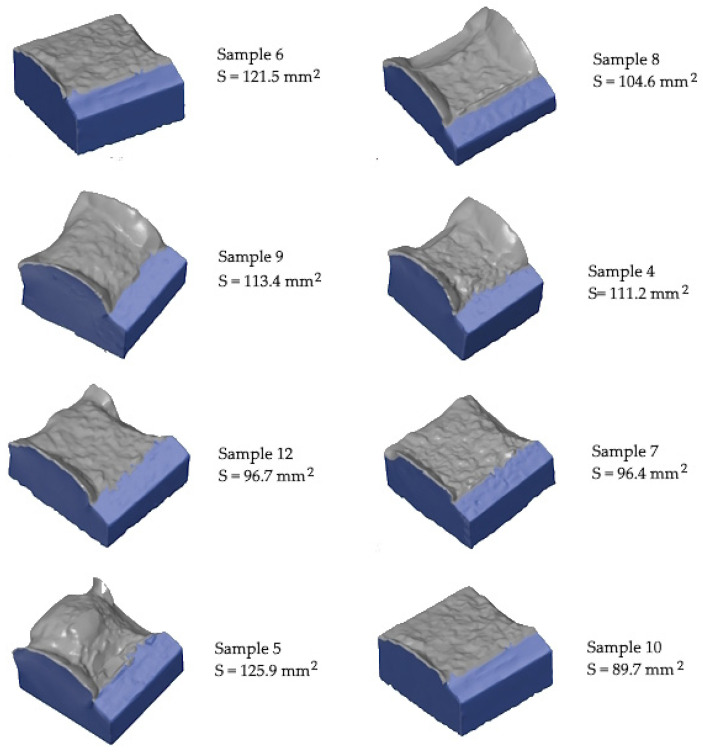
Fracture surface of Charpy test specimens; the fracture surface areas were measured.

To further understand the significant differences in impact toughness values, 3D fracture surface morphology after Charpy impact testing was characterized using 3D optical scanner. Figure 14 presents the fracture surface of sample (No. 4, 5, 6, 7, 8, 9, 10, and 12) specimens. In all specimens, the fracture initiated from the root of the notch due to stress concentration. For all samples, the two kinds of surfaces, the pure cracking, and the shear lip surfaces can be observed. The shear lips were formed as the material deforms due to shear stress. In the case of the 20 µm layer thickness, all fracture surfaces reveal significant and relatively symmetric shear lip regions.

**Figure 15:**
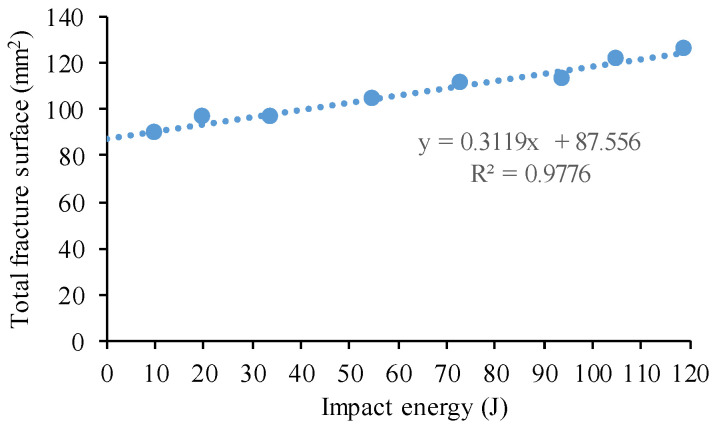
Plot of impact energy vs. total fracture surface.

A big neck is formed at the fracture surface in low porosity samples. The surface (S) of the cracking and shear lip regions was measured for the selected specimens. Figure 15 shows the relationship between the absorbed energy and S (total fracture surface). A good linear relation can be seen between the fracture surface and the impact energy. To quantitatively prove the linear correlation, the correlation coefficient was calculated. The value of the correlation coefficient is very close to 1, indicating a total positive linear correlation between the absorbed Charpy impact energy and the 3D fracture surface area.

## 4. Conclusions

Laser-based metal additive manufacturing has the potential to be applied in the production of parts for safety-critical applications (e.g., aircraft components, armor and military applications), where materials must be able to absorb energy before breaking. Charpy impact testing is a standardized method used to assess the toughness of metals. The general aim of the present study was to understand the effect of power level on the porosity evolution and material performance. The impact fracture strength of maraging steel 1.2709 was investigated. The following conclusions could be drawn:

1. Highly dense samples were achieved, and the experiment results show that a relative density of 99.97% could be achieved for 1.2709 using the LPBF technique;
2. The optimal process parameters for the lowest porosity were P = 150 W, v = 600 mm/s, and t = 0.02 mm;
3. A porosity below 0.25% has almost no effect on the resulting tensile properties;
4. Porosity has a significant influence on impact energy, even for samples with low porosity, and the impact energy drops abruptly as porosity increases; therefore, impact testing may be used for parameter optimization;
5. The maximum absorbed energy value achieved was 119 J. When comparing this result with the highest-impact energy values of as-built LPBF 1.2709 specimens available in the literature, an improvement of more than 20% was achieved;
6. A linear relationship between the Charpy impact energy and the inverse of porosity was found;
7. A linear correlation between the Charpy impact energy and the area of 3D fracture surface was observed.

## Figures and Tables

**Table 1:** Charpy and porosity test results for 1.2709 specimens.

Sample	P	v	t	VED	Impact Energy	Porosity
	W	mm/s	mm	J/mm^3^	J	%
1	125	400	0.02	156.3	51	0.24
2	150	400	0.02	187.5	20	0.7
3	175	400	0.02	218.8	12	1.96
4	125	600	0.02	104.2	73	0.11
5	150	600	0.02	125.0	119	0.03
6	175	600	0.02	145.8	105	0.07
7	125	800	0.02	78.1	20	0.6
8	150	800	0.02	93.8	55	0.13
9	175	800	0.02	109.4	94	0.05
10	125	1000	0.02	62.5	10	1.69
11	150	1000	0.02	75.0	18	0.37
12	175	1000	0.02	87.5	34	0.26
13	125	400	0.04	78.1	13	3.3
14	150	400	0.04	93.8	23	1.74
15	175	400	0.04	109.4	23	1.19
16	125	600	0.04	52.1	11	1.22
17	150	600	0.04	62.5	45	0.21
18	175	600	0.04	72.9	66	0.11
19	125	800	0.04	39.1	8	4.01
20	150	800	0.04	46.9	13	0.65
21	175	800	0.04	54.7	27	0.25
22	125	1000	0.04	31.3	6	11.48
23	150	1000	0.04	37.5	8	3.56
24	175	1000	0.04	43.8	13	1.19

## Data Availability

The original contributions presented in the study are included in the article, further inquiries can be directed to the corresponding author.
